# Circulating Tumor Cells in Uveal Melanoma: Multi-Marker Detection and Association With Disease State

**DOI:** 10.1167/iovs.67.1.55

**Published:** 2026-01-27

**Authors:** Daniel P. de Bruyn, Fabiana Lucia Bassil, Mike Wu, Aaron B. Beasley, Jolanda Vaarwater, Mai N. Van, Jaco Kraan, Robert M. Verdijk, Dion Paridaens, Caroline M. van Rij, Nicole C. Naus, Annelies de Klein, Elin S. Gray, Erwin Brosens, Emine Kiliç

**Affiliations:** 1 Department of Ophthalmology, Erasmus MC, The Netherlands; 2 Department of Clinical Genetics, Erasmus MC, The Netherlands; 3 Centre for Precision Health, School of Medical and Health Sciences, Edith Cowan University, Joondalup, Australia; 4 Department of Clinical Oncology, Erasmus MC, The Netherlands; 5 Department of Pathology, Section Ophthalmic Pathology, Erasmus MC, The Netherlands; 6 Department of Ophthalmic Pathology, Leiden University Medical Centre, Leiden, The Netherlands; 7 The Eye Hospital, Rotterdam, The Netherlands; 8 Department of Radiotherapy, Erasmus MC, The Netherlands

**Keywords:** circulating tumor cells, liquid biopsy, uveal melanoma, prognostication, noninvasive

## Abstract

**Purpose:**

Uveal melanoma (UM) is primarily treated with eye-sparing radiotherapy, leaving limited tumor tissue for molecular analysis. Circulating tumor cells (CTCs) may offer a minimally invasive alternative for genomic tumor profiling. This pilot study evaluated the feasibility of a multi-marker CTC capture approach in UM patients at diagnosis, during fractionated stereotactic radiotherapy (fSRT), and at metastatic progression.

**Methods:**

Patients with localized or metastatic UM were prospectively enrolled. Peripheral blood samples were collected at baseline, during fSRT, and on detection of metastases. CTCs were captured and enumerated using a multi-marker approach and fluorescence microscopy.

**Results:**

A total of 76 patients were included: 68 with localized disease and eight with metastatic disease. Four patients presented initially with localized disease but developed metastasis during follow-up: for comparisons, only metastatic-stage samples were used, yielding 64 localized and 12 metastatic samples. CTCs were detected in 69.1% of patients with localized disease at baseline and in 83.3% of those with metastases. CTC counts were significantly higher in metastatic disease than in localized disease (median = 8 vs. 3; *P* = 0.010). Among patients undergoing fSRT, paired analysis showed a significant increase in CTC counts between day 3 and day 5 (median = 1 vs. 4; *P* = 0.007). No significant associations were observed between baseline CTC counts and tumor thickness, largest basal diameter, tumor volume, American Joint Committee on Cancer tumor stage, tumor location, or molecular risk class.

**Conclusions:**

Multi-marker CTC detection in UM patients is feasible across disease stages. Increased CTC counts during fSRT may offer a window for molecular characterization. Larger studies with longitudinal blood sampling are needed to validate clinical and prognostic utility.

Uveal melanoma (UM) is the most common primary intraocular tumor, accounting for approximately 5% of all melanomas, with an age-adjusted incidence of 5.1 cases per million individuals annually.^1^^–^^3^ UM occurs predominantly in the choroid (90%), followed by the ciliary body (6%) and the iris (4%).^4^

Prognostication in UM has shifted from relying mostly on clinical and histopathological characteristics to incorporating molecular markers, with chromosomal and mutational profiles providing the strongest predictors of metastatic risk.^5^^–^^10^ Loss of chromosome 3 and inactivating mutations in *BRCA1 associated protein 1* (*BAP1*) are strongly associated with early metastatic spread and reduced progression-free survival, with most patients developing metastases within five years after diagnosis.^7^^,^^11^ In contrast, hotspot mutations in *splicing factor 3b subunit 1* (*SF3B1*), often coinciding with disomy 3, are associated with a bimodal pattern of metastasis, with peaks at approximately five and eight years after diagnosis.^12^^,^^13^ Tumors with a mutated *eukaryotic translation initiation factor 1A, X-linked* (*EIF1AX*) generally have the best prognosis, as these seldom metastasize.^12^^,^^14^

Advances in radiotherapy and earlier detection have shifted treatment of UM from primarily enucleations to mostly eye-sparing therapies, such as fractionated stereotactic radiotherapy (fSRT), plaque brachytherapy, and proton beam therapy.^15^ Although prognostic biopsies are now routinely and safely performed in many specialized ocular oncology centers for individual risk stratification, their use is not yet a universal standard of care across institutions.^16^^–^^18^ Despite long-term evidence supporting the feasibility and clinical utility of techniques such as fine-needle aspiration biopsy, decisions regarding tumor sampling remain influenced by patient-, tumor-, and center-specific factors, including tumor size and location, local expertise, and procedural considerations.^16^^–^^18^ In addition, intraocular biopsies typically rely on single-site sampling, which may fail to capture the genetic heterogeneity of the tumor, potentially leading to the misclassification of metastatic risk.^16^^,^^19^^,^^20^ In particular, obtaining adequate tissue yield can be challenging in small or posterior tumors, and biopsy-related complications, although generally infrequent, remain an important clinical consideration.^16^^,^^17^ Meanwhile, systemic therapies for metastatic UM are rapidly evolving. Tebentafusp, a gp100-HLA directed CD3 T-cell engager, has demonstrated improved survival in metastatic UM, particularly when tumor burden is low, and is currently being evaluated in the adjuvant setting.^21^ These developments highlight the critical need for minimally invasive biomarkers that can provide molecular and prognostic information when tumor tissue cannot be obtained.^22^

Liquid biopsies offer promising minimally invasive alternatives for molecular characterization in UM, as they circumvent the risks and limitations of intraocular tumor biopsy and enable real-time monitoring of tumor dynamics.^15^^,^^19^^,^^23^ Circulating tumor DNA (ctDNA) can quantify tumor load and monitor minimal residual disease (MRD) using chromosomal and mutational profiles.^24^^–^^26^ Reliable ctDNA detection in UM depends strongly on tumor size and disease stage, and remains challenging in patients with localized disease, as ctDNA levels are often undetectable or very low in early-stage tumors.^27^^–^^29^

Circulating tumor cells (CTCs), shed from the primary or metastatic tumors into the bloodstream, offer a valuable complementary liquid biopsy approach by providing intact viable cells for comprehensive genomic, transcriptomic, and proteomic characterization.^23^^,^^30^^–^^32^ This is especially relevant in UM where tumor heterogeneity may influence metastatic potential and treatment response.^23^^,^^31^ CTCs have been detected in both localized and metastatic UM, although reported detection rates vary depending on the enrichment strategy and capture markers.^15^^,^^31^^–^^33^ Early studies using single markers most likely underestimated CTC numbers because of heterogeneous antigen expression characteristic of UM.^31^ Recent strategies using multiple capture markers or marker-independent microfluidic and immunomagnetic methods have improved detection sensitivity and reproducibility.^31^^,^^32^ Furthermore, treatment-induced tumor matrix disruption, such as during radiotherapy, may transiently increase CTC shedding and offer opportunities to assess dynamic changes in CTC levels.^34^ Few studies have systematically examined these dynamics across different disease phases, with most previous CTC studies focusing on single time points, limiting understanding of how CTC levels change throughout disease progression and treatment.^35^^–^^37^

In this pilot study, we applied a multi-marker immunomagnetic CTC capture approach in UM patients at diagnosis, during fSRT, and at time of metastasis detection. By evaluating CTC dynamics across different disease phases, we aimed to assess the feasibility of this multi-marker CTC detection, characterize temporal CTC fluctuations, and explore associations with known prognostic factors.

## Methods

### Uveal Melanoma Cohort

Patients were prospectively enrolled in this study between 2019 and 2024, following ethical approval of the Erasmus MC medical ethics committee (MEC-2009-375). Written informed consent was obtained from all patients, and the study adhered to the tenets of the declaration of Helsinki. Patients were recruited from the Rotterdam Ocular Melanoma Study group and included those with untreated primary UM or metastatic UM. Patients with iris melanomas were excluded, because these tumors behave differently than choroidal UMs and exhibit distinct genotypic features.^38^ UMs originating in the ciliary body and extending into the iris were included. For each patient, demographic and clinical information were collected, including age, sex, date of diagnosis, baseline tumor characteristics (e.g., tumor size), primary treatment, presence and, if applicable, date of metastases, date of blood collection, and any co-occurring malignancies. The standard clinical workup included ophthalmologic screening by an ocular oncologist with ultrasound imaging of the tumor, and metastatic surveillance via chest radiography, abdominal ultrasound scanning, and blood tests (lactate dehydrogenase and g-glutamyltransferase). UM was staged according to The American Joint Committee on Cancer (AJCC) Staging Manual 8th edition based on ultrasound measurements.^39^ Tumor volume was estimated using the formula previously described by Stålhammar et al.,^40^ which uses the largest basal diameter and tumor thickness. Primary treatment consisted of enucleation, proton beam therapy, brachytherapy, or fSRT. For patients treated with radiation, secondary tumor resection was performed in case of a high risk of developing toxic tumor syndrome. Patients treated with fSRT between 2019 and 2024 received in-house treatment using the Cyberknife stereotactic radiosurgery system consisting of 10 Gray (Gy) per day for five consecutive days. Patients treated with proton beam therapy or brachytherapy were treated at an external facility, which limited blood sample collection during their treatment.

### Blood Collection

Peripheral blood samples were collected from all patients at baseline (time of diagnosis) and—when applicable—at the time of detection of metastases. For patients treated with fSRT, additional blood samples were collected on days 1, 3, and 5 of treatment. Blood was drawn into 10 mL Streck cell-free DNA blood collection tubes (Streck, La Vista, NE, USA), which preserve CTCs for up to seven days at room temperature and processed within 48 hours for centrifugation and CTC extraction. Plasma was separated from the blood using centrifugation as previously described.^31^

### Circulating Tumor Cell Retrieval and Enumeration

The CTC capturing protocol was adapted from Beasley et al.,^31^ with the substitution of magnetic-activated-cell-sorting (MACS) beads (Miltenyi Biotec, Bergisch Gladbach, Germany) for Dynabeads (ThermoFisher Scientific, Waltham, MA, USA). MACS MicroBeads are smaller (30–100 nm), do not interfere with downstream fluorescent staining and microscopy, and allow more efficient CD45-depletion and subsequent multi-marker enrichment compared to larger Dynabeads (1–5 µm), which can interfere with imaging and cell analysis.^15^^,^^41^ Peripheral blood mononuclear cells were isolated from 10 mL of peripheral blood by density gradient centrifugation using Ficoll-Paque Plus (Cytiva, Uppsala, Sweden) in 50 mL Leucosep tubes (Greiner Bio-One, Kremsmünster, Austria). The resulting cell pellet was resuspended in 500 µL of MACS buffer (PBS with 0.5% BSA, and 2 mM EDTA). Cells were first incubated with 10 µL CD45 MACS microbeads (Miltenyi Biotec) for 15 minutes at 4°C. CD45-positive cells were subsequently depleted using magnetic LD-columns (Miltenyi Biotec) on a QuadroMACS (Miltenyi Biotec) separator. The remaining CD-negative cell suspension was washed with MACS buffer, then incubated for 30 minutes at 4°C with a mixture of 20 µL Fc receptor blocking agent (Miltenyi Biotec), 10 µL CD146-bound MACS beads (Miltenyi Biotec), 10 µL anti-melanoma (MCSP)-bound MACS microbeads (Miltenyi Biotec), 0.5 µg gp100-biotin antibody (Novus Biologicals, Englewood, CO, USA), 1.0 µg ATP Binding Cassette Subfamily B Member 5–biotin (Novus Biologicals) antibody solution. After washing with MACS buffer, cells were incubated with anti-biotin MACS microbeads (Miltenyi Biotec) for 15 minutes at 4°C. Positively labeled cells were isolated using magnetic MS-columns (Miltenyi Biotec) on a miniMACS separator (Miltenyi Biotec). After magnetic enrichment, cells were washed twice with MACS buffer and fixed with Fix & Perm medium A (ThermoFisher Scientific) for 10 minutes at room temperature.

**Figure 1.**
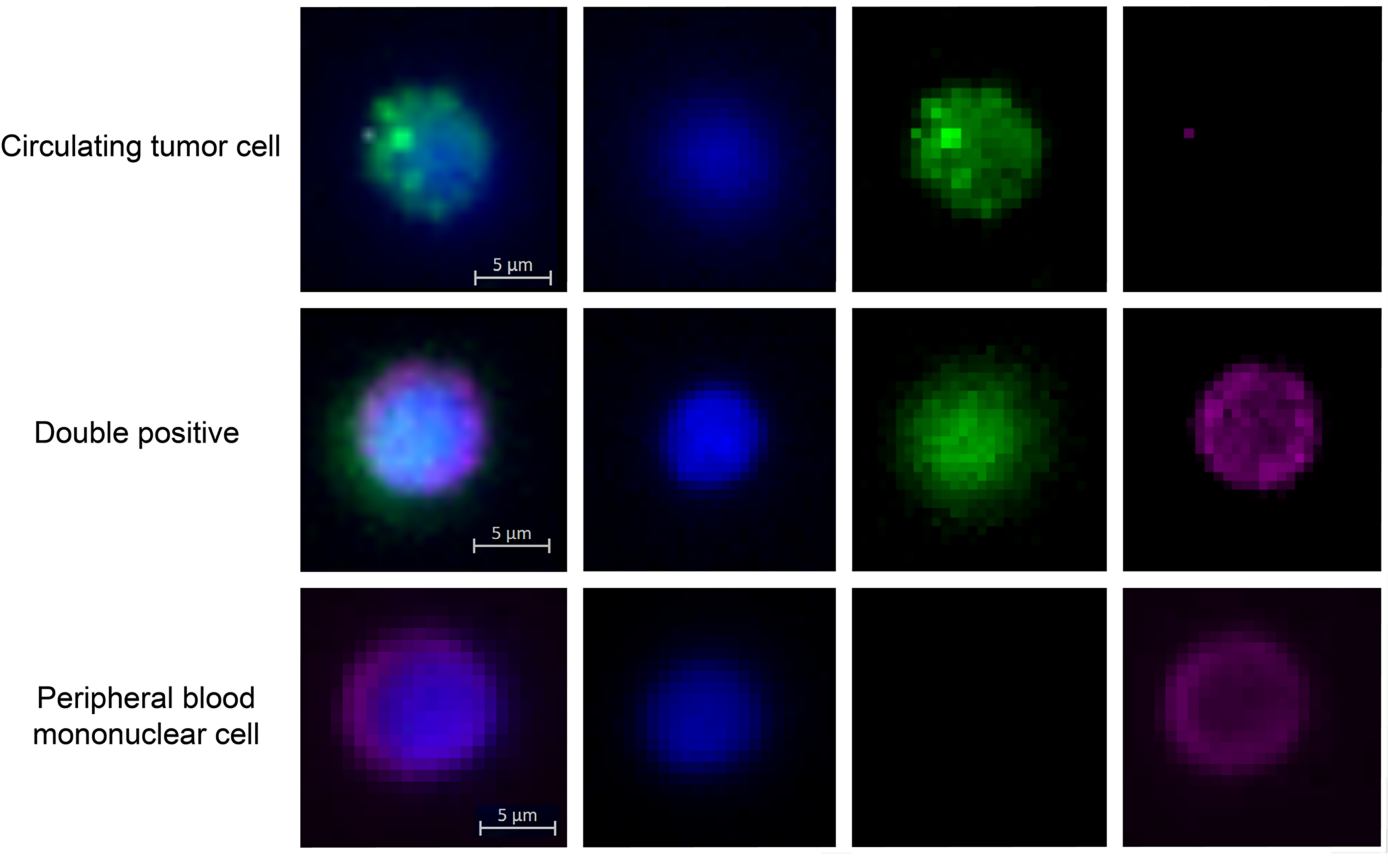
Example of a circulating tumor cell, a “double positive,” and peripheral blood mononuclear cell. Cells were classified as CTCs when they were round (circularity = 0.75) and had nuclear staining (Hoechst positive) with positive staining for MART1, gp100, or S100B (FITC-positive) and negative staining for CD16 and CD45 (Cy5-negative). A double-positive cell was classified as a peripheral blood mononuclear cell that is positive for Hoechst, FITC, and Cy5. A peripheral blood mononuclear cell is characterized by a positive Hoechst and CD16/CD45 staining.

Fixed cells were incubated in 5% FcR blocking agent for 10 minutes at room temperature, then permeabilized and incubated for one hour with Fix & Perm medium B and 2% normal donkey serum, 1:50 anti-MelanA (Abcam, Cambridge, UK), 1:50 anti-melanoma gp100 (Abcam), 1:500 anti-s100b (ThermoFisher Scientific), 5 µL anti-CD16-Alexa Fluor (AF) 647 (BioLegend, San Diego, CA, USA), and 5 µL anti-CD45-AF647 (BioLegend). After washing, cells were incubated again in Fix & Perm medium B with 2% normal donkey serum, 1:500 donkey anti-rabbit IgG AF488 (ThermoFisher Scientific), and 10 µg/mL Hoechst 33342 (Invitrogen, Waltham, MA, USA). Cells were washed twice with PBS mounted on adhesion microscope slides (SlideMate Plus; Epredia, Kalamazoo, MI, USA) using Dako fluorescence mounting medium (Agilent, Santa Clara, CA, USA). Whole-slide images were obtained using a Zeiss Axio Imager Z2 (Carl Zeiss AG, Oberkochen, Germany) with a 10× objective and motorized stage. Images were analyzed using FIJI ImageJ.^42^ CTCs were defined as round cells (circularity ≥ 0.75) with Hoechst positive nucleated cells, FITC positive staining (gp100, MART1 and s100b), and Cy5 negative (CD45 and CD16) staining (Fig. 1).

### Molecular Analysis

Molecular information was retrieved when tumor tissue was available and included histopathological assessment, mutation analysis, and copy number variation (CNV) profiling. Diagnosis of UM and assessment of metastatic risk were confirmed by an experienced ocular oncology pathologist (RV) when sufficient tumor material was available, as previously described.^7^ Briefly, histopathological markers, including cell type, lymphocytic infiltration, mitotic rate, necrosis, ciliary body involvement, and extraocular extension were assessed on formalin-fixed paraffin-embedded tissue sections stained with hematoxylin and eosin-stained. The presence of extracellular matrix loops was evaluated on periodic acid-Schiff–stained slides using a green filter. BAP1 protein expression was determined by BAP1 immunohistochemistry on formalin-fixed paraffin-embedded sections, as previously described.^7^ DNA for primary and secondary driver mutation analysis DNA was extracted from tumor tissue and analyzed using a custom gene panel on the IonTorrent platform, as previously described.^43^ Libraries were prepared using the Ampliseq library kit (ThermoFisher Scientific) and sequenced with the Ion Genestudio 5 using a custom panel including *GNAQ, GNA11, PLCB4, CYSLTR2, BAP1, SF3B1, EIF1AX, SRSF2, U2AF1*, and *MBD4*. CNV profiling was performed using the Illumina Infinium Global Screening Array-24 v3.0 kit (Illumina, San Diego, CA, USA), as previously described.^43^ In short, 200ng of DNA was used for the screening array and resulting IDAT files were processed using Genome Studio v2.0 (Illumina). CNV profiles were generated by comparing sample data to the Illumina reference dataset and visualized using Nexus Copy Number (version 10.1; BioDiscovery, El Segundo, CA, USA).

### Metastatic Risk Stratification

Metastatic risk was stratified based on the genetic and protein expression profiles of the primary tumor, which predict both the likelihood and timing of metastatic spread.^6^ In patients without available primary tumor material, but with metastatic tissue, molecular profiling of the metastasis was used, as the driver mutations are highly concordant between primary and metastatic UM.^44^^,^^45^ Based on these profiles, tumors with loss of BAP1 protein expression and/or an inactivating *BAP1* mutation were classified as high risk. Tumors harboring a *SF3B1* mutation while retaining BAP1 protein expression were classified as intermediate risk. Tumors with retained BAP1 protein expression and either an *EIF1AX* mutation or a favorable CNV profile were classified as low risk.^43^ Because risk stratification was not possible when no tumor tissue was available for molecular testing, these cases were classified as unknown.

### Statistical Analysis

Statistical analyses were performed using R 4.5.0 (R Foundation for Statistical Computing, Vienna, Austria) and RStudio version 2023.12.0.369 (Posit Software; PBC, Boston, MA, USA). Overall follow-up time was defined as the time from diagnosis to death or censoring. Patients who were alive at the time of analysis were censored at their last known follow-up date. Follow-up time was calculated in months using the date of diagnosis and either the date of death or last follow-up. Two-sided tests were used throughout, and a *P* value <0.05 was considered statistically significant.

## Results

### Cohort Description

A total of 76 patients were prospectively included. At diagnosis, all patients had localized UM. Blood samples at localized disease were available for 68 patients, with baseline blood available for 65 samples. During follow-up, four of these patients developed metastases, and additional blood samples were collected at the time of metastatic disease. For three patients, blood was only collected during fSRT treatment for localized disease. In another eight patients, no baseline sample was available, but blood was collected at the time of metastatic disease.

The median age at diagnosis was 69.70 years [IQR range 61.63-74.80] for patients with localized disease and 67.30 years [IQR range 65.46-75.24] for those with metastatic disease. The localized group included 41 males and 23 females, whereas the metastatic group included six males and six females. Among patients with localized disease, primary tumor treatment consisted mostly of fSRT (*n* = 31), followed by proton beam therapy (*n* = 18), enucleation (*n* = 12), and brachytherapy (*n* = 3). In the metastatic group, most primary tumors were treated with either fSRT (*n* = 5) or proton beam therapy (*n* = 5), followed by enucleation (*n* = 2). Mean overall follow-up time was 31.99 ± 16.50 months for patients with localized disease and 46.89 ± 21.75 months for those with metastatic disease. The follow-up duration was longer in patients who developed metastases compared to those with localized disease, as they were already under surveillance for localized disease and continued to be monitored after progression. Consequently, for most of these patients, blood samples were only available at the time of metastasis.

Risk classification proved feasible in 40 patients. For 37 of them, genetic and molecular data were available from the primary tumor, while metastatic tissue was available in the remaining three. For 33 of these patients, blood samples collected at baseline were available, allowing comparison of CTC counts across molecular risk class. Most patients in both the localized and metastatic groups were classified as high metastatic risk based on their molecular profile. For the remaining 36 patients, no primary tissue was available, and their molecular risk class was therefore categorized as “Unknown,” although some later developed metastases. More extensive patient and tumor characteristics are summarized in the Table and Supplementary Table S1.

**Table.** Patient Characteristics for the Localized and Metastatic Disease Groups

Characteristics	Localized	Metastatic^*^
Number of patients	64	12
Sex		
Male	41	6
Female	23	6
Median age at onset [IQR]	69.70 [61.63–74.80]	67.30 [65.46–75.24]
Metastatic risk class		
High risk	18 (28.1%)	8 (66.7%)
Intermediate risk	8 (12.5%)	0
Low risk	6 (9.4%)	0
Unknown	32 (50%)	4 (33.3%)
Primary therapy		
fSRT	31 (48.4%)	5 (41.7%)
Brachytherapy	3 (4.7%)	0
Enucleation	12 (18.8%)	2 (16.7%)
Proton beam therapy	18 (28.1%)	5 (51.7%)
Mean largest basal diameter ± SD (mm), range^†^	12.76 ± 3.95 (4.70–22.70)	14.28 ± 1.97 (10.60–16.60)
Mean thickness ± SD (min-max) (mm), range^†^	8.53 ± 13.11 (0.50–17.10)	9.72 ± 3.49 (3.90–14.50)
Mean volume ± SD (min-max) (mm^3^), range^‡^	635.99 ± 597.99 (28.8–2361.8)	928.26 ± 415.70 (213.90–1471.70)
Tumor location		
Choroid	55 (85.9%)	12 (100%)
Ciliary body	9 (14.1%)	0
AJCC Tumor stage		
1	15 (23.4%)	0
2	17 (26.6%)	2 (16.7%)
3	21 (32.8%)	9 (75%)
4	11 (17.2%)	1 (8.3%)
Mean overall follow-up time ± SD (months)	31.99 ± 16.50	46.89 ± 21.75

AJCC, American Joint Committee on Cancer; fSRT, fractionated stereotactic radiotherapy; IQR, interquartile range; SD, standard deviation.

* The four patients who developed metastases during follow-up are included in the metastatic group, as baseline characteristics reflect the primary tumors that later progressed.

† Tumor size (largest basal diameter and tumor thickness) was evaluated by ultrasound of the primary tumor at time of diagnosis.

‡ Tumor volume was estimated using the formula previously described by Stålhammar et al.^40^

### CTC Count in Disease State and Treatment

Counts of recovered CTCs per patient and timepoint of blood collection are shown in Supplementary Table S2. Baseline blood samples obtained before treatment (fSRT, proton beam therapy, brachytherapy, or enucleation) were available for 65 patients with localized disease. CTCs were detected in 47 of 65 (69.1%) patients with localized disease at baseline and in 10 of 12 (83.3%) patients with metastatic disease.

**Figure 2.**
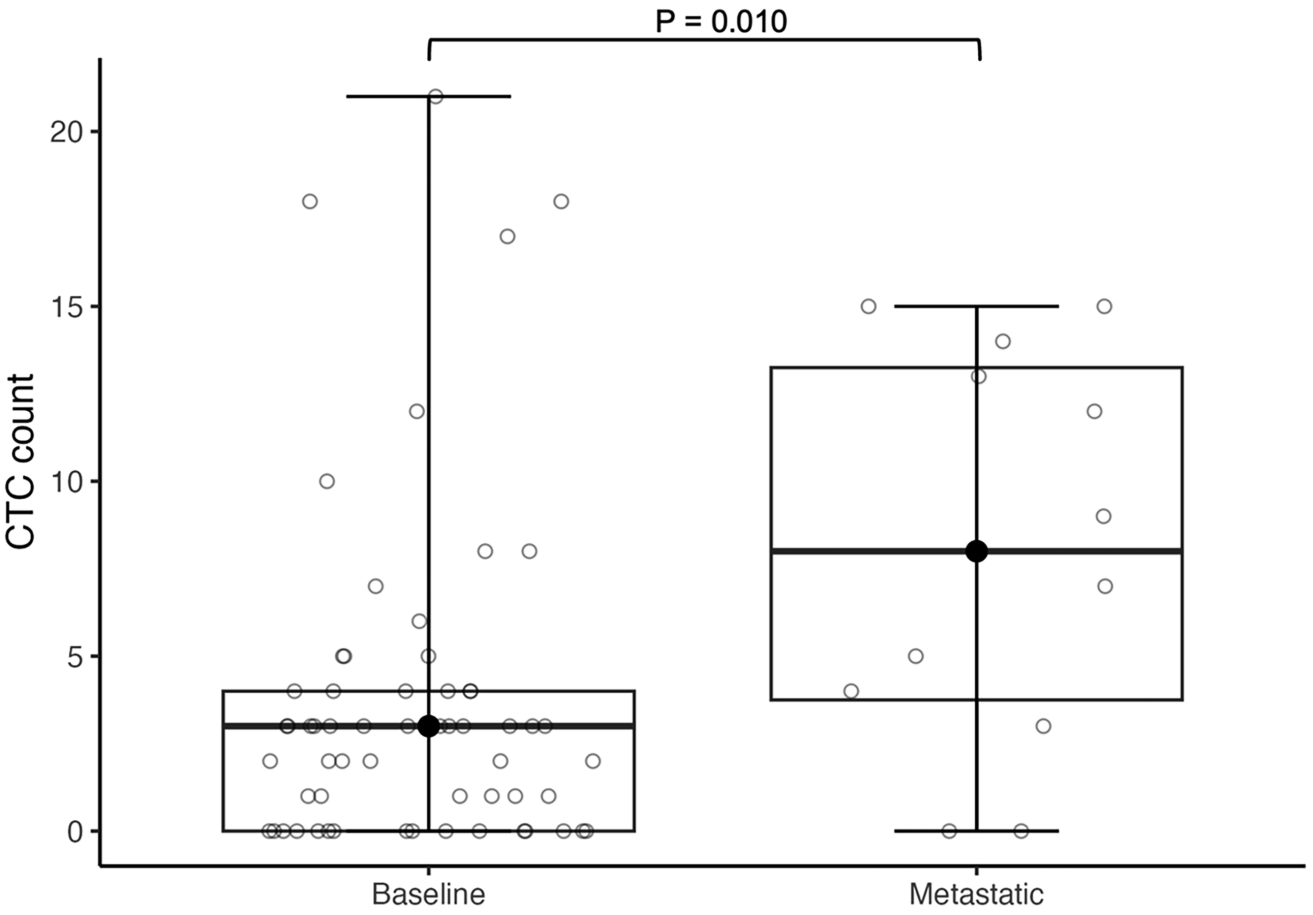
CTC counts in localized and metastatic uveal melanoma. Significantly higher CTC counts were observed in patients with metastatic disease (*n* = 12; median 8; min 0 – max 15) compared to those with localized disease (*n* = 61; median 3; min 0 – max 21) (*P* = 0.010, W = 194.5 Wilcoxon rank-sum test). *Boxplots* display the IQR; the *bold horizontal line with the black dot* indicates the median; the whiskers represent the minimum and maximum values.

In the comparison between localized and metastatic disease, only the metastatic samples from the four patients who developed metastases during follow-up were included in order to maintain independent groups and increase the metastatic sample size. Patients with metastatic disease (*n* = 12) had a higher number of recovered CTCs (median = 8; range 0-15) compared to patients with localized disease (*n* = 61; median = 3; range 0-21) (W = 194.5, *P* = 0.010, Wilcoxon rank-sum test) (Fig. 2).

**Figure 3.**
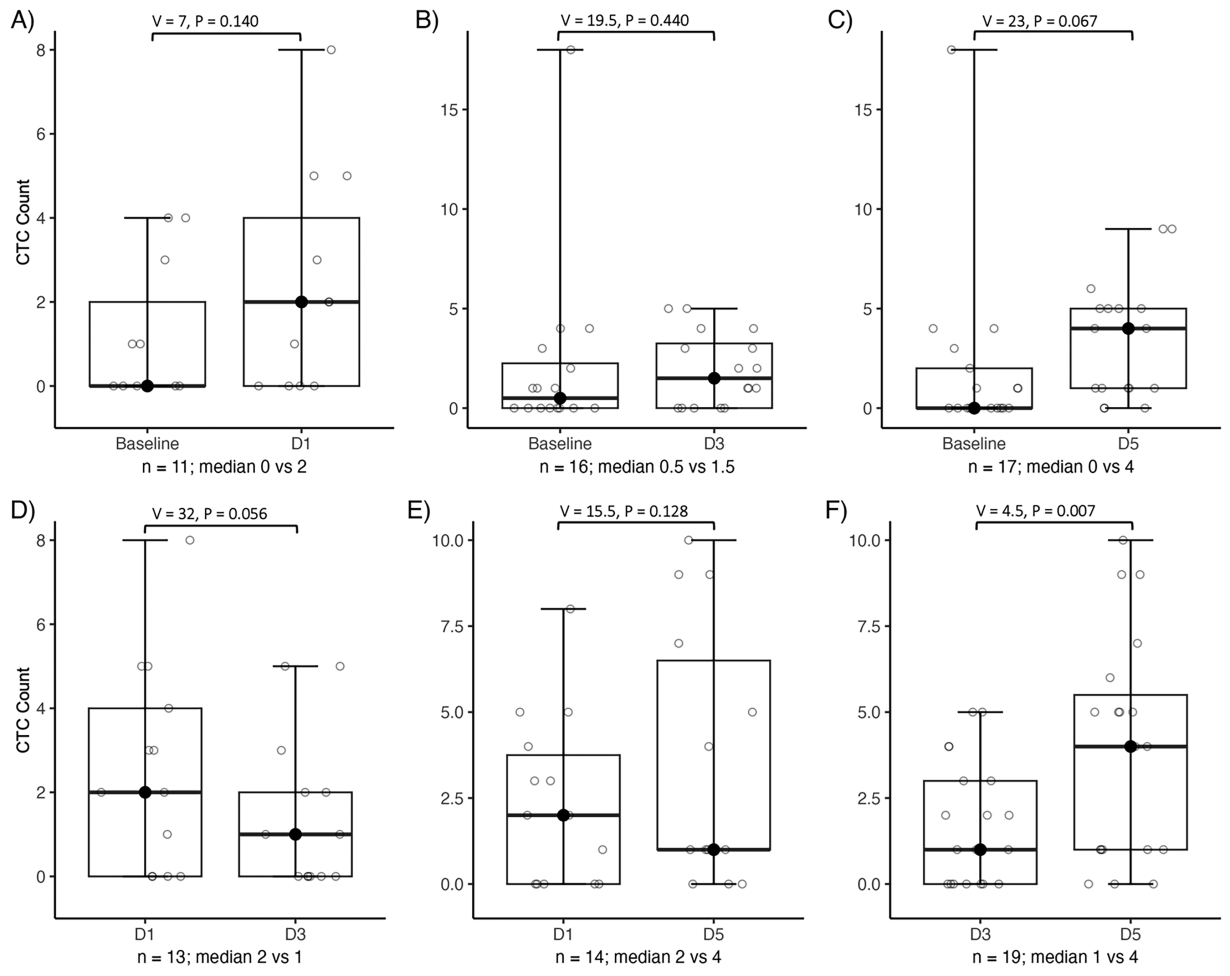
CTC counts across the various days of fSRT. Paired comparisons were performed using the Wilcoxon signed-rank test. **(A–C)** No significant differences were observed between baseline and any of the fSRT timepoints. **(D, E)** No significant differences were found between day 1 (D1) and day 3 (D3), or between D1 and day 5 (D5) of fSRT. **(F)** A significant increase in CTC count was observed between D3 and D5 of fSRT (V = 4.5, *P* = 0.007). *Boxplots* display the IQR; the *bold horizontal line with the black dot* indicates the median; the *whiskers* represent the minimum and maximum values.

In patients with localized disease undergoing fSRT, paired blood samples collected at multiple timepoints during fSRT allowed evaluation of radiation-induced changes in CTC counts using the paired Wilcoxon signed-rank test (Fig. 3; Supplementary Table S2; Supplementary Fig. S1). No statistically significant changes were observed between baseline and day 1 (*n* = 11; median = 0 [range 0-4] vs. 2 [0-8]; *P* = 0.140, V = 7), baseline and day 3 (*n* = 16; 0.5 [0-18] vs. 1.5 [0-5]; *P* = 0.440, V = 19.5), or baseline and day 5 (*n* = 17; 0 [0-18] vs. 4 [0-9]; *P* = 0.067, V = 23). Similarly, no significant differences were found between day 1 and day 3 (*n* = 13; 2 [0-8] vs. 1 [0-5]; *P* = 0.056, V = 32) or between day 1 and day 5 (*n* = 14; 2 [0-8] vs. 1 [0-10]; *P* = 0.128, V = 15.5). However, between day 3 and day 5, CTC counts increased significantly from a median of 1 (range 0–5) to median 4 (range 0–10) (*n* = 19; *P* = 0.007, V = 4.5) (Fig. 3F).

### CTC Analysis

**Figure 4.**
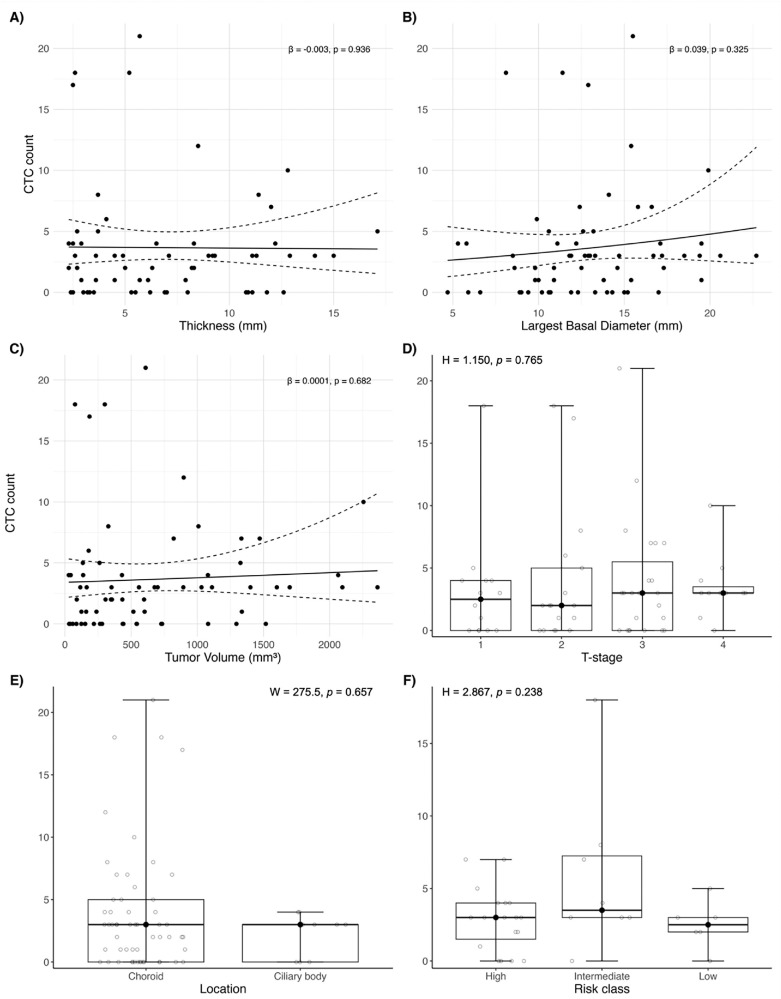
Tumor characteristics and CTC counts in localized disease. **(A)** Tumor thickness (Negative binomial regression *β* = 0.039, *P* = 0.325) and **(B)** largest basal diameter (β = −0.003, *P* = 0.936) did not significantly correlate with the number of recovered CTCs. **(C)** Larger tumor volume was not significantly associated with higher baseline CTC counts (*β* = 0.0001, *P* = 0.682). **(D)** CTC counts did not differ significantly between AJCC tumor categories (H = 1.150, *P* = 0.765; Kruskal-Wallis test). **(E)** No significant difference in CTC was observed between tumors located in the choroid and those in the ciliary body (W = 275.5, *P* = 0.657; Wilcoxon rank sum test). **(F)** The absolute CTC count did not significantly differ among high-, intermediate-, and low-risk tumors (H = 2.867, *P* = 0.239; Kruskal-Wallis test). The *solid lines* in panels **A–C** represent the fitted regression line, with the dashed lines indicating the 95% confidence interval. In panels **D–F**, boxplots display the IQR, the *bold horizontal line* with the *black dot* indicates the median, and the *whiskers* represent the minimum and maximum values.

All available baseline samples (*n* = 65) were included in the analyses assessing associations between CTC counts and tumor characteristics. Negative binomial regression showed no significant association between tumor thickness and baseline CTC count (*β* = −0.003, *P* = 0.936) or largest basal diameter and baseline CTC count (*β* = 0.039, *P* = 0.325) (Figs. 4A, 4B). Similarly, larger tumor volume was not significantly associated with higher baseline CTC counts (*β* = 0.0001, *P* = 0.682) (Fig. 4C). The number of detected CTCs at baseline did not differ significantly between AJCC T-stages (H = 1.150, *P* = 0.765; Kruskal-Wallis test) (Fig. 4D). Median CTC counts were similar for tumors located in the ciliary body and choroid, both with a median of 3 CTCs (range 0–4 and 0-21, respectively; W = 275.5, *P* = 0.657; Wilcoxon rank sum test) (Fig. 4E). Among the 33 patients with known metastatic risk and available baseline blood samples, CTC counts did not differ significantly across risk groups (H = 2.867, *P* = 0.238; Kruskal-Wallis) (Fig. 4F).

## Discussion

CTC detection and characterization have emerged as highly promising tools in cancer diagnostics and monitoring, offering valuable understanding of tumor biology, prognosis, and dissemination. In several tumor types, including breast and prostate cancer, CTCs are already used as liquid biomarkers.^31^^,^^46^^,^^47^ In UM, however, their application is still emerging. In this study, we demonstrate the feasibility of multi-marker CTC enumeration in UM and show that patients with metastatic UM have significantly higher CTC counts compared to those with localized disease, consistent with previous studies.^35^^,^^36^^,^^48^

Early detection of metastases is important for timely enrollment into clinical trials when metastatic lesions are still small.^21^ In our study, CTCs were detected in 69% of patients with localized disease at baseline and in 83% of patients with metastases, underscoring the feasibility of CTC enumeration across disease stages. In an earlier study, Beasley et al.^36^ detected CTCs in 58% of patients (15 of 26 patients) with primary UM using a protocol based solely on MCSP capture. Suesskind et al.,^37^ using a single-marker method found even lower CTC counts in localized disease (14%). Subsequent refinement of the Beasley protocol in 2021 to a multi-marker protocol targeting MCSP, CD146, gp100 and ATP-binding cassette sub-family 5 resulted in a markedly improved recovery rate of 86% (37 of 43 patients) in localized UM.^31^ Other reported detection rates in UM vary widely from 14% to 68%, largely reflecting methodological differences such as enrichment strategies, marker selection, and blood volume^33^^,^^35^^,^^49^ Multi-marker approaches generally achieve higher recovery than single-marker methods, highlighting the importance of antibody selection and multi-marker strategies.

In our study, CTCs were not detected in approximately 30% of patients with primary UM and 20% of metastatic cases. The absence of detectable CTCs highlights both technical and biological limitations. Biologically, sampling may coincide with minimal or intermittent tumor cell release. While technically, limited blood volume, assay sensitivity, and antigen heterogeneity may contribute to false-negative results.^32^ Thus, although CTCs are a promising liquid biopsy source in UM, absence of detection does not exclude tumor activity, and improved enrichment and detection methodologies are needed.

Beasley et al.^36^ also showed potential for genomic characterization, as they demonstrated that CTCs from a patient with metastatic UM harbored chromosomal aberrations nearly identical to the excised primary tumor. Despite these advances, downstream molecular characterization of isolated CTCs remains challenging. In our hands, low-pass sequencing yielded inconsistent results, due to technical limitations, including sequencing artifacts and inconsistent detection of chromosomal aberrations. Recently, Grisanti et al.^33^ successfully determined chromosomal status in CTCs from UM using FISH, enabling visual confirmation of high- and low-risk genomic profiles. However, in our attempts to replicate this approach, FISH did not yield in interpretable results. This may be explained by key methodological differences: unlike Grisanti et al.,^33^ who processed large blood volumes (∼50 mL) in a single isolation step and enzymatically released captured cells for cytospin-based mounting, we processed smaller volumes (∼10 mL) without bead release. Additionally, their use of methanol-based post-fixation likely optimized nuclear accessibility for hybridization, whereas our FIX & PERM-based protocol may have limited probe penetration. Moreover, the low number of recovered CTCs in localized disease further constrained molecular analysis. To our knowledge, Grisanti et al.^33^ remain the only group to have successfully applied FISH on CTCs in UM to date.

CTCs were identified here using immunophenotyping, specifically selecting for cells positive for gp100/MART1/S100β and negative for CD45/CD16, to ensure melanoma-specific detection and minimize contamination from non-melanoma hematopoietic cells.^31^^,^^36^^,^^50^ Because of the workflow used for CTC retrieval—which includes fixation of the enriched cells for downstream staining and imaging—viable CTCs are not preserved. As a result, ex vivo culture or expansion of CTCs was not possible in our study, and functional assessment or confirmation of their tumor-initiating capacity could not be performed. Although short-term culture of CTCs from UM patients has been reported, long-term expansion and functional validation remain technically challenging and are not yet routine in the field.^23^^,^^32^^,^^50^^,^^51^ Future research using dedicated viable-cell isolation methods or single-cell expansion strategies may help determine whether UM CTCs possess tumor-initiating capacity and further clarify their biological relevance.^23^

To improve detection sensitivity, CTC counts were evaluated at multiple timepoints during fSRT. We observed a statistically significant increase in CTCs between day three and day five of radiation, whereas changes between baseline and other timepoints were not statistically significant. The rise in CTCs on the last day of fSRT may reflect radiation-induced mobilization of tumor cells, consistent with earlier findings in other solid tumors, where radiotherapy disrupts the tumor microenvironment and promotes CTC shedding into the circulation.^34^ The biological relevance of this transient increase remains uncertain. Although treatment-related cellular release is plausible, sampling effects cannot be excluded. Notably, only ten patients had complete sampling across all timepoints, limiting interpretation and underscoring the need for confirmation in larger cohorts. Suesskind et al.^37^ found no significant difference in CTC counts before and after various primary therapies, suggesting that therapy does not consistently lead to detectable CTC shedding into the systemic circulation. However, their methodology was limited to pre- and post-treatment sampling and may have missed transient intratreatment changes in CTC levels. Consistent with our observations, recent studies in UM and other solid tumors report acute increases of CTCs or ctDNA during radiotherapy in the circulation, supporting a true biological effect.^28^^,^^29^^,^^52^ This increase in CTCs during treatment may provide a valuable window for downstream molecular analyses. Although recent evidence demonstrates that tissue-based prognostic biopsies can be reliably performed after radiotherapy,^53^ in our center tissue for primary genotyping after radiotherapy is often unavailable. Moreover, tissue availability and testing success may remain constrained by factors such as DNA yield, timing after treatment, and sampling limitations.^53^ Consequently, liquid biopsy approaches such as CTC and ctDNA analysis remain particularly attractive. Analyzing the genetic cargo of CTCs could help determine metastatic risk and be used for prognostication.^15^ Moreover, molecular characterization of CTCs may uncover therapeutic options targeting metastases.^54^ However, low CTC counts in localized disease remain a major limiting factor for such analyses.^36^^,^^55^

Although UM is a rare disease, our cohort of 68 patients with localized disease is relatively large. However, the number of metastatic patients was limited (*n* = 12), which may have reduced the statistical power of subgroup analyses. Still, we observed significantly higher CTC counts in patients with metastases, consistent with observations by Anand et al.^35^ In this study, elevated CTC levels in metastatic patients were measured at a time when metastases were already clinically detectable, therefore, CTC enumeration cannot be interpreted as predictive in this setting. To determine whether rising CTC counts can identify metastatic progression earlier than imaging, longitudinal sampling throughout follow-up would be required.

No significant associations were found between CTC counts and tumor location, AJCC T-stage, or metastatic risk class. Genetic data were available for 32 patients with localized disease, the majority of whom had tumors with a high metastatic risk-profile, possibly reflecting a selection bias, as high-risk tumors with *BAP1* mutations or chromosome 3 loss tend to be larger and are more often surgically removed.^56^ Although no statistically significant differences were observed, subgroup analyses were limited by small sample sizes, reducing statistical power and increasing the likelihood of type II errors, indicating that subtle biological effects cannot be excluded. Previous studies have reported higher CTC counts in patients with high-risk tumors and larger lesions, supporting the biological relevance of CTCs as liquid biopsy markers.^31^^,^^33^^,^^35^^,^^48^

An important avenue for future exploration is the relationship between CTC counts and metastatic tumor volume. In a subset of patients with available PET imaging of the metastasis, metabolic tumor volume was previously shown to correlate significantly with ctDNA levels.^24^ A similar correlation with CTC count could be meaningful in detecting metastases. Our preliminary observations suggest a significant correlation between higher amounts of CTCs and the presence of metastasis. Further studies incorporating volumetric PET or MRI measurements alongside CTC levels could provide deeper insight into the dynamics of tumor shedding and help define thresholds for clinical utility.

## Conclusions

In summary, our study demonstrates the feasibility of CTC detection in UM using a multi-marker enrichment approach. Furthermore, it highlights the importance of sampling timing and technique in maximizing recovery. CTC recovery increased significantly during fSRT, particularly between day 3 and day 5, offering a potential window for downstream characterization. Our findings also show that patients with metastatic UM have significantly higher CTC counts compared to those with localized disease. Further research with longitudinal sampling is needed to evaluate whether CTC dynamics can precede radiologic detection, to explore associations with tumor burden, and to validate their clinical and prognostic utility in larger cohorts.

## Supplementary Material

Supplement 1
