# Supplementary material for: Circulating Tumor Cells in Uveal Melanoma: Multi-Marker Detection and Association With Disease State
Source: Invest Ophthalmol Vis Sci. 2026 Jan 27;67(1):55. doi: 10.1167/iovs.67.1.55 (PMC12859728; doi:10.1167/iovs.67.1.55)
Supplement: Supplement 1 [file iovs-67-1-55_s001.pdf]

# Supplementary Materials

Circulating Tumor Cells in Uveal Melanoma: Multi-Marker Detection and Association with Disease State

D.P. de Bruyn[,1,2,†], F. L. Bassil[1,2,†], M. Wu[1,2], A.B. Beasley[3], J. Vaarwater[1], N. Van[4], J. Kraan[4], R.M. Verdijk[5,6], D. Paridaens[7], C.M. van Rij[8], N.C. Naus[1], A. de Klein[2], E.S. Gray[3], E. Brosens[2$], and E. Kiliç[1$]

1 Department of Ophthalmology, Erasmus MC, The Netherlands
2 Department of Clinical Genetics, Erasmus MC, The Netherlands
3 Centre for Precision Health, School of Medical and Health Sciences, Edith Cowan University, Joondalup, Western Australia, Australia
4 Department of Clinical Oncology, Erasmus MC, The Netherlands
5 Department of Pathology, section Ophthalmic Pathology, Erasmus MC
6 Department of ophthalmic pathology Leiden University Medical Centre
7 The Eye Hospital, Rotterdam
8 Department of Radiotherapy, Erasmus MC, The Netherlands

[†] These authors contributed equally to this work.
[$] These authors share last authorship

**Index**

**Supplementary Table 1.** Characteristics of patients included in this study.
**Supplementary Table 2.** Recovery of circulating tumor cells (CTCs) per patient.
**Supplementary Figure 1.** Recovery of circulating tumor cells (CTCs) during fractioned stereoradiotherapy (fSRT) per patient.

**Supplementary Table 1.** Characteristics of patients included in this study. Tumor T-stage according to the AJCC tumor classification was assessed by ultrasound [1]. Risk class is defined by the combination of copy number variation-patterns, mutated secondary driver gene and BAP1 protein expression[2].

| | Disease stage | Age at onset | FU (months) | Tumor location | T-stage | LBD (mm) | Thickness (mm) | Risk class | Primary driver | Mutation | Secondary driver | Mutation | Chromosome status | Bap1 staining | Primary therapy |
|---|---|---|---|---|---|---|---|---|---|---|---|---|---|---|---|
| **P1*** | Metastatic | 67.31 | 87.3 | Choroid | 2 | 11.1 | 3.9 | High | GNA11 | c.626_627delA GinsTT; p.GLN209Leu | BAP1 | c.1838delC; p.Thr613Argfs*4 | NA | NA | fSRT |
| **P2** | Metastatic | 68.11 | 67.1 | Choroid | 3 | 16.4 | 7.2 | High | GNAQ | c.626A>C; p.Q209P | BAP1 | c.1310_1029del; p.L143Hfs*7 | Monosomy 3 | Absent | fSRT |
| **P3** | Metastatic | 79.36 | 72.4 | Choroid | 3 | 13.4 | 9.7 | NA | NA | NA | NA | NA | NA | NA | fSRT |
| **P4** | Metastatic | 64.95 | 33 | Choroid | 4 | 15.6 | 12.8 | High | GNAQ | c.626A>C; p.Q209P | BAP1 | c.-3_15del; p.? | Monosomy 3; 8q gain | Absent | Enucleation |
| **P5** | Metastatic | 65.63 | 68.1 | Choroid | 3 | 16.1 | 6.4 | High | GNA11 | c.626A>T; p.Q209L | BAP1 | c.68-2A>G | Monosomoy 3; 6p gain; 8q gain | Absent | Enucleation |
| **P6** | Localized | 61.56 | 41.4 | Choroid | 2 | 9.8 | 8.3 | High | PLCB4 | c.1889A>G | BAP1 | c.1878del; p.K626Nfs*11 | Monosomy 3; 8q gain | Absent | Enucleation |
| **P7** | Localized | 70.35 | 63.2 | Choroid | 1 | 8.1 | 2.6 | NA | NA | NA | NA | NA | NA | NA | fSRT |
| **P8** | Localized | 70.00 | 62.8 | Choroid | 2 | 10 | 3.6 | NA | NA | NA | NA | NA | NA | NA | fSRT |
| **P9** | Localized | 66.77 | 63.9 | CB | 3 | 15 | 10.8 | NA | NA | NA | NA | NA | NA | NA | fSRT |
| **P10** | Metastatic | 50.04 | 36 | Choroid | 3 | 13.5 | 11.3 | High | NA | NA | BAP1 | c.1313_1319del; p.S438Cfs*131 | NA | Absent | fSRT |
| **P11** | Localized | 60.95 | 37.5 | Choroid | 2 | 13.6 | 5.3 | High | GNAQ | c.626A>C; p.Q209P | BAP1 | c.947_954del; p.A316Vfs*79 | Monosomy 3; 8q gain | Absent | fSRT |
| **P12** | Localized | 70.77 | 51.3 | Choroid | 2 | 10.9 | 8.2 | Low | GNAQ | c.626A>C; p.Q209P | EIF1AX | c.7A>G; p.K3E | 6p gain | Present | fSRT |
| **P13** | Localized | 61.65 | 51.3 | Choroid | 3 | 15.4 | 8.5 | NA | NA | NA | NA | NA | NA | NA | fSRT |
| **P14** | Localized | 77.33 | 43.3 | Choroid | 4 | 19.9 | 12.8 | NA | NA | NA | NA | NA | NA | NA | PBT |
| **P15** | Localized | 67.37 | 52.3 | Choroid | 1 | 8.6 | 2.7 | NA | NA | NA | NA | NA | NA | NA | PBT |
| **P16** | Localized | 78.02 | 42.6 | CB | 3 | 11.8 | 11.3 | High | GNA11 | c.626A>T; p.Q209L | BAP1 | c.5C>T; p.P2L | Monosomy 3; 8q gain | Absent | fSRT |
| **P17** | Localized | 50.62 | 22.4 | Choroid | 3 | 12.3 | 10.9 | Low | GNA11 | c.626A>T; p.Q209L | NA | NA | 6p gain; 8q gain | Present | Enucleation |
| **P18** | Localized | 42.82 | 32.1 | Choroid | 2 | 11.9 | 7 | Interme diate | GNAQ | c.626A>T; p.Q209L | BAP1_VUS /SF3B1 | c.1874G>A; p.R625H | 6p gain; 8q gain | Present | PBT |

| | | | | | | | | | | | | | | | |
|---|---|---|---|---|---|---|---|---|---|---|---|---|---|---|---|
| **P19** | Metastatic | 79.04 | 42.4 | Choroid | 3 | 13.6 | 14.5 | High | GNAQ | c.626A>C; p.Q209P | BAP1 | c.799_800del; p.Q267Afs*16 | Monosomy 3; 8q gain | Absent | PBT |
| **P20** | Localized | 64.16 | 27 | Choroid | 3 | 12.4 | 12 | Intermediate | GNAQ | c.626A>C; p.Q209P | SF3B1 | c.1874G>A; p.R625H | 6p gain; 8q gain | Present | fSRT |
| **P21** | Metastatic | 67.28 | 40.5 | Choroid | 3 | 14.4 | 13.6 | NA | NA | NA | NA | NA | NA | NA | PBT |
| **P22** | Localized | 77.66 | 37.2 | Choroid | 3 | 13.3 | 6.9 | High | GNAQ | c.626A>C; p.Q209P | BAP1 | deletion exon 4 | Monosomy 3; 8q gain | Absent | fSRT |
| **P23** | Localized | 69.92 | 45 | Choroid | 1 | 5.9 | 2.5 | NA | NA | NA | NA | NA | NA | NA | fSRT |
| **P24** | Localized to metastatic | 66.15 | 23 | Choroid | 2 | 10.6 | 5.3 | NA | NA | NA | NA | NA | NA | NA | PBT |
| **P25** | Localized | 59.13 | 48.1 | Choroid | 2 | 10.2 | 3.3 | NA | NA | NA | NA | NA | NA | NA | fSRT |
| **P26** | Localized | 72.09 | 50 | Choroid | 1 | 5.8 | 2.9 | NA | NA | NA | NA | NA | NA | NA | PBT |
| **P27** | Localized | 70.23 | 49.1 | Choroid | 1 | 6.6 | 3 | NA | NA | NA | NA | NA | NA | NA | fSRT |
| **P28** | Localized | 74.62 | 49.5 | CB | 1 | 4.7 | 3.2 | NA | NA | NA | NA | NA | NA | NA | fSRT |
| **P29** | Localized | 70.21 | 44.3 | Choroid | 4 | 13.2 | 17.1 | High | GNAQ | c.626A>C; p.Q209P | BAP1 | deletion exon 1-3 | Monosomy 3 | Absent | PBT |
| **P30** | Localized | 62.00 | 39.5 | Choroid | 3 | 15.4 | 5.7 | NA | NA | NA | NA | NA | NA | NA | PBT |
| **P31** | Localized | 74.92 | 37.7 | Choroid | 1 | 9.8 | 2.9 | NA | NA | NA | NA | NA | NA | NA | fSRT |
| **P32*** | Localized | 55.74 | 39.7 | Choroid | 4 | 15.2 | 12.6 | High | NA | NA | BAP1 | NA | NA | NA | PBT |
| **P33** | Localized | 50.72 | 17.2 | Choroid | 1 | 5.3 | 2.3 | NA | NA | NA | NA | NA | NA | NA | Brachytherapy |
| **P34** | Localized | 83.57 | 0.8 | Choroid | 3 | 17.1 | 8.3 | High | GNA11 | c.626A>T; p.Q209L | BAP1 | c.375+2T>C | Monosomy 3; 8q gain | Absent | PBT |
| **P35** | Localized | 61.91 | 45.3 | Choroid | 4 | 18.5 | 9.2 | NA | NA | NA | NA | NA | NA | NA | PBT |
| **P36** | Localized | 70.36 | 45.8 | Choroid | 1 | 6.3 | 3 | NA | NA | NA | NA | NA | NA | NA | fSRT |
| **P37** | Localized to metastatic | 74.75 | 39.2 | Choroid | 3 | 14.3 | 8 | NA | NA | NA | NA | NA | NA | NA | PBT |
| **P38** | Localized | 86.14 | 19.9 | Choroid | 3 | 13.8 | 6.1 | NA | NA | NA | NA | NA | NA | NA | fSRT |
| **P39** | Localized | 78.03 | 33.1 | Choroid | 3 | 14.6 | 6.3 | NA | NA | NA | NA | NA | NA | NA | fSRT |
| **P40** | Localized | 78.81 | 17.5 | Choroid | 1 | 9 | 2.4 | NA | NA | NA | NA | NA | NA | NA | fSRT |
| **P41** | Localized | 53.01 | 31.6 | CB | 4 | 19.5 | 12.2 | High | GNA11 | c.626A>T; p.Q209L | BAP1 | c.1773_1795del; p.S592Gfs*43 | Monosomy 3 | Absent | PBT |
| **P42** | Localized | 76.43 | 36.4 | Choroid | 2 | 14.3 | 2.4 | NA | NA | NA | NA | NA | NA | NA | fSRT |

| | | | | | | | | | | | | | | | |
|---|---|---|---|---|---|---|---|---|---|---|---|---|---|---|---|
| **P43** | Localized | 71.98 | 3.7 | Choroid | 4 | 17.2 | 12.9 | Intermediate | GNAQ | c.626A>C; p.Q209P | SF3B1 | c.1874G>A; p.R625H | Monosomy 3; 6p gain; 8q gain | Present | PBT |
| **P44** | Localized | 61.32 | 4.8 | Choroid | 2 | 12.7 | 4.9 | NA | NA | NA | NA | NA | NA | NA | Brachytherapy |
| **P45** | Localized | 71.70 | 40.9 | CB | 3 | 13 | 9 | High | GNAQ | c.626A>G; p.Q209R | BAP1 | c.539T>C; p.180P | Monosomy 3 | Absent | Enucleation |
| **P46** | Localized | 70.75 | 38.4 | CB | 3 | 12.2 | 6.5 | High | GNAQ | c.626A>T; p.Q209L | BAP1 | deletion exon 5 | Monosomy 3; 8q gain | Absent | fSRT |
| **P47** | Localized | 78.56 | 38.4 | Choroid | 1 | 11.9 | 2.6 | NA | NA | NA | NA | NA | NA | NA | PBT |
| **P48** | Localized | 57.55 | 16.6 | CB | 4 | 20.6 | 11.1 | High | GNA11 | c.626A>T; p.Q209L | BAP1 | c.122+5G>A | Monosomy 3; 8q gain | Absent | Enucleation |
| **P49** | Localized to metastatic | 76.72 | 16.6 | CB | 3 | 15.8 | 12 | High | GNAQ | c.626A>C; p.Q209P | BAP1 | c.1671_1684del; p.S558Afs*4 | Monosomy 3; 8q gain | Absent | PBT |
| **P50** | Localized | 65.96 | 34.5 | CB | 3 | 9.4 | 11.1 | NA | NA | NA | NA | NA | NA | NA | fSRT |
| **P51 *** | Localized | 57.73 | 14.5 | Choroid | 2 | 10.7 | 5.5 | High | GNAQ | c.626A>c; p.Q209P | BAP1/EIF1AX_VUS | c.256-2A>T & c.5C>A; p.P2H | Monosomy 3 | NA | fSRT |
| **P52** | Localized | 51.74 | 36.4 | CB | 4 | 16.7 | 12.9 | High | GNAQ & PLCB4 | c.548G>A; p.R183Q & c.1888G>A; p. D630N | MBD4 | c.1402C>T; p. R468W | Monosomy 3 | Absent | PBT |
| **P53** | Localized | 61.95 | 28.7 | Choroid | 2 | 14.1 | 3.7 | NA | NA | NA | NA | NA | NA | NA | fSRT |
| **P54** | Localized to metastatic | 61.84 | 32.3 | Choroid | 3 | 16.6 | 12 | High | GNAQ | c.626A>T; p.Q209L | BAP1 | c.1153C>T; p.R385* | Monosomy 3; 8q gain | Absent | fSRT |
| **P55** | Localized | 65.98 | 35.3 | Choroid | 2 | 10.9 | 4.5 | NA | NA | NA | NA | NA | NA | NA | fSRT |
| **P56** | Localized | 61.93 | 26.8 | Choroid | 1 | 8.9 | 3.5 | NA | NA | NA | NA | NA | NA | NA | fSRT |
| **P57** | Localized | 54.74 | 21.6 | Choroid | 4 | 19.5 | 7.9 | High | GNA11 | c.626A>T; p.Q209L | BAP1 | c.587G>A; p.W196* | Monosomy 3; 8q gain | Absent | Enucleation |
| **P58** | Localized | 75.79 | 0.6 | Choroid | 3 | 12.9 | 15 | Intermediate | NA | NA | SF3B1 | c.1874G>A; p.R625H | 8q gain | Present | Enucleation |
| **P59** | Localized | 61.91 | 31 | Choroid | 3 | 13.3 | 7.1 | High | GNA11 | c.626A>T; p.Q209L | BAP1 | c.659+3A>T | Monosomy 3; 6p gain | Absent | Enucleation |
| **P60** | Localized | 64.29 | 28.3 | Choroid | 4 | 22.7 | 4.5 | Low | GNAQ | c.626A>T; p.Q209L | NA | NA | 6p gain; 8q gain | Present | Enucleation |
| **P61** | Localized | 58.63 | 25.8 | Choroid | 3 | 15.5 | 5.7 | NA | NA | NA | NA | NA | NA | NA | PBT |
| **P62** | Localized | 76.79 | 21.6 | Choroid | 3 | 10.3 | 12.6 | NA | NA | NA | NA | NA | NA | NA | fSRT |
| **P63** | Localized | 54.77 | 25.6 | Choroid | 3 | 14 | 6.2 | NA | NA | NA | NA | NA | NA | NA | PBT |
| **P64** | Localized | 72.86 | 9 | Choroid | 2 | 12.9 | 2.5 | NA | NA | NA | NA | NA | NA | NA | Brachytherapy |

| | | | | | | | | | | | | | | | |
|---|---|---|---|---|---|---|---|---|---|---|---|---|---|---|---|
| **P65** | Localized | 74.76 | 26.7 | Choroid | 2 | 11.4 | 5.2 | Intermediate | GNAQ | c.626A>G; p.Q209R | SF3B1 | c.1972T>C; p.W658R | 6p gain; 8q gain | Present | fSRT |
| **P66** | Localized | 70.18 | 14.2 | Choroid | 2 | 12.6 | 3.7 | NA | NA | NA | NA | NA | NA | NA | fSRT |
| **P67** | Localized | 65.75 | 8.6 | Choroid | 2 | 9.9 | 4.1 | NA | NA | NA | NA | NA | NA | NA | fSRT |
| **P68** | Localized | 58.44 | 1.4 | Choroid | 1 | 10.6 | 2.7 | Low | GNA11 | c.626A>T; p.Q209L | EIF1AX | c.25G>C; p.G9R | NA | Present | PBT |
| **P69** | Localized | 67.98 | 14.8 | Choroid | 2 | 17.3 | 2.3 | High | GNA11 | c.626A>T; p.Q209L | BAP1 | c.382G>A; p.G128R | Monosomy 3; 8q gain | Absent | Enucleation |
| **P70** | Localized | 69.48 | 26.2 | Choroid | 3 | 17 | 11.8 | High | GNA11 | c.626A>T; p.Q209L | BAP1 | c.510dup; p.V171Cfs*12 | Monosomy 3; 8q gain | Absent | PBT |
| **P71** | Localized | 78.12 | 14.7 | Choroid | 1 | 11.1 | 2.5 | Intermediate | GNAQ | c.626A>C; p.Q209P | SF3B1 | c.1874G>A; p.R625H | NA | NA | fSRT |
| **P72** | Localized | 81.95 | 2.7 | Choroid | 4 | 19.4 | 14.1 | Intermediate | GNAQ | c.626A>C; p.Q209P | SF3B1 | c.1873C>T; p.R625C | 6p gain; 8q gain | Present | Enucleation |
| **P73** | Localized | 75.11 | 12.6 | Choroid | 2 | 12.5 | 5 | Low | GNA11 | c.626A>T; p.Q209L | EIF1AX | c.16G>C; p.G6R | NA | Present | fSRT |
| **P74** | Localized | 84.38 | 14.7 | Choroid | 3 | 14.1 | 11.4 | Intermediate | GNA11 | c.626A>T; p.Q209L | SF3B1 | c.1874G>T; p.R625L | 6p gain; 8q gain | Present | Enucleation |
| **P75** | Localized | 73.48 | 2.7 | Choroid | 1 | 8.5 | 3.6 | Low | GNAQ | c.626A>T; p.Q209L | NA | NA | NA | Present | PBT |
| **P76** | Localized | 63.77 | 12.6 | Choroid | 3 | 14.7 | 9.3 | High | NA | NA | BAP1 | c.1883C>G, p.S628* | Monosomy 3 | Absent | Enucleation |

Abbreviations: FU: follow-up time; CB: ciliary body; LBD: largest basal diameter; fsrt: fractionated stereotactic radiotherapy; PBT: proton beam therapy; NA: not available.
***** Molecular risk class was determined using metastatic tissue.

**Supplementary Table 2.** Recovery of circulating tumor cells (CTCs) per patient. CTCs were recovered prior to at diagnosis (baseline), on day 1 (D1), day 3 (D3), or day 5 (D5) of fractioned stereotactic radiotherapy and when metastases (metastasis) were present.

| Patient | Baseline | D1 | D3 | D5 | Metastasis | Risk class |
|---|---|---|---|---|---|---|
| **P1** | | | | | 5 | High |
| **P2** | | | | | 15 | High |
| **P3** | | | | | 12 | Unknown |
| **P4** | | | | | 9 | High |
| **P5** | | | | | 7 | High |
| **P6** | 2 | | | | | High |
| **P7** | 18 | | 4 | 5 | | Unknown |
| **P8** | 1 | 0 | 0 | 0 | | Unknown |
| **P9** | 0 | | 4 | 5 | | Unknown |
| **P10** | | | | | 13 | High |
| **P11** | | 0 | 0 | 1 | | High |
| **P12** | 2 | | 2 | 6 | | Low |
| **P13** | 12 | | | | | Unknown |
| **P14** | 10 | | | | | Unknown |
| **P15** | 2 | | | | | Unknown |
| **P16** | 3 | 0 | 0 | 0 | | High |
| **P17** | 0 | | | | | Low |
| **P18** | 0 | | | | | Intermediate |
| **P19** | | | | | 14 | High |
| **P20** | 7 | | | | | Intermediate |
| **P21** | | | | | 15 | Unknown |
| **P22** | 0 | 2 | 3 | 1 | | High |
| **P23** | 0 | 2 | 0 | 4 | | Unknown |
| **P24** | 0 | | | | 0 | Unknown |
| **P25** | 0 | | 0 | 4 | | Unknown |
| **P26** | 4 | | | | | Unknown |
| **P27** | 0 | 0 | | 1 | | Unknown |
| **P28** | 0 | 0 | 1 | 1 | | Unknown |
| **P29** | 5 | | | | | High |
| **P30** | 1 | | | | | Unknown |
| **P31** | 1 | | 1 | 1 | | Unknown |
| **P32** | 0 | | | | | High |
| **P33** | 4 | | | | | Unknown |
| **P34** | 4 | | | | | High |
| **P35** | 3 | | | | | Unknown |
| **P36** | | 4 | 0 | 10 | | Unknown |
| **P37** | 0 | | | | 0 | Unknown |
| **P38** | 1 | 1 | 1 | 1 | | Unknown |
| **P39** | 2 | | | | | Unknown |
| **P40** | 0 | 5 | 2 | 9 | | Unknown |
| **P41** | 4 | | | | | High |
| **P42** | 0 | 3 | 0 | 0 | | Unknown |

| | | | | | | |
|---|---|---|---|---|---|---|
| **P43** | 3 | | | | | Intermediate |
| **P44** | 3 | | | | | Unknown |
| **P45** | 3 | | | | | High |
| **P46** | 4 | 5 | 5 | 9 | | High |
| **P47** | 3 | | | | | Unknown |
| **P48** | 3 | | | | | High |
| **P49** | 7 | | | | 4 | High |
| **P50** | 0 | | 3 | 5 | | Unknown |
| **P51** | 0 | | | | | High |
| **P52** | 3 | | | | | High |
| **P53** | 8 | | | | | Unknown |
| **P54** | 7 | | | | 3 | High |
| **P55** | 1 | | | | | Unknown |
| **P56** | 0 | | | | | Unknown |
| **P57** | 1 | | | | | High |
| **P58** | 3 | | | | | Intermediate |
| **P59** | 3 | | | | | High |
| **P60** | 3 | | | | | Low |
| **P61** | 21 | | | | | Unknown |
| **P62** | | 3 | 2 | 7 | | Unknown |
| **P63** | 0 | | | | | Unknown |
| **P64** | 17 | | | | | Unknown |
| **P65** | 18 | | | | | Intermediate |
| **P66** | 5 | | | | | Unknown |
| **P67** | 6 | | | | | Unknown |
| **P68** | 5 | | | | | Low |
| **P69** | 2 | | | | | High |
| **P70** | 0 | | | | | High |
| **P71** | 4 | 8 | 5 | 5 | | Intermediate |
| **P72** | 3 | | | | | Intermediate |
| **P73** | 2 | | | | | Low |
| **P74** | 8 | | | | | Intermediate |
| **P75** | 3 | | | | | Low |
| **P76** | 3 | | | | | High |

**Supplementary Figure 1.** Recovery of circulating tumor cells (CTCs) during fractionated stereoradiotherapy (fSRT) per patient. The risk groups are shown in different line colors. The dashed lines represent patients that developed metastasis at a later timepoint.

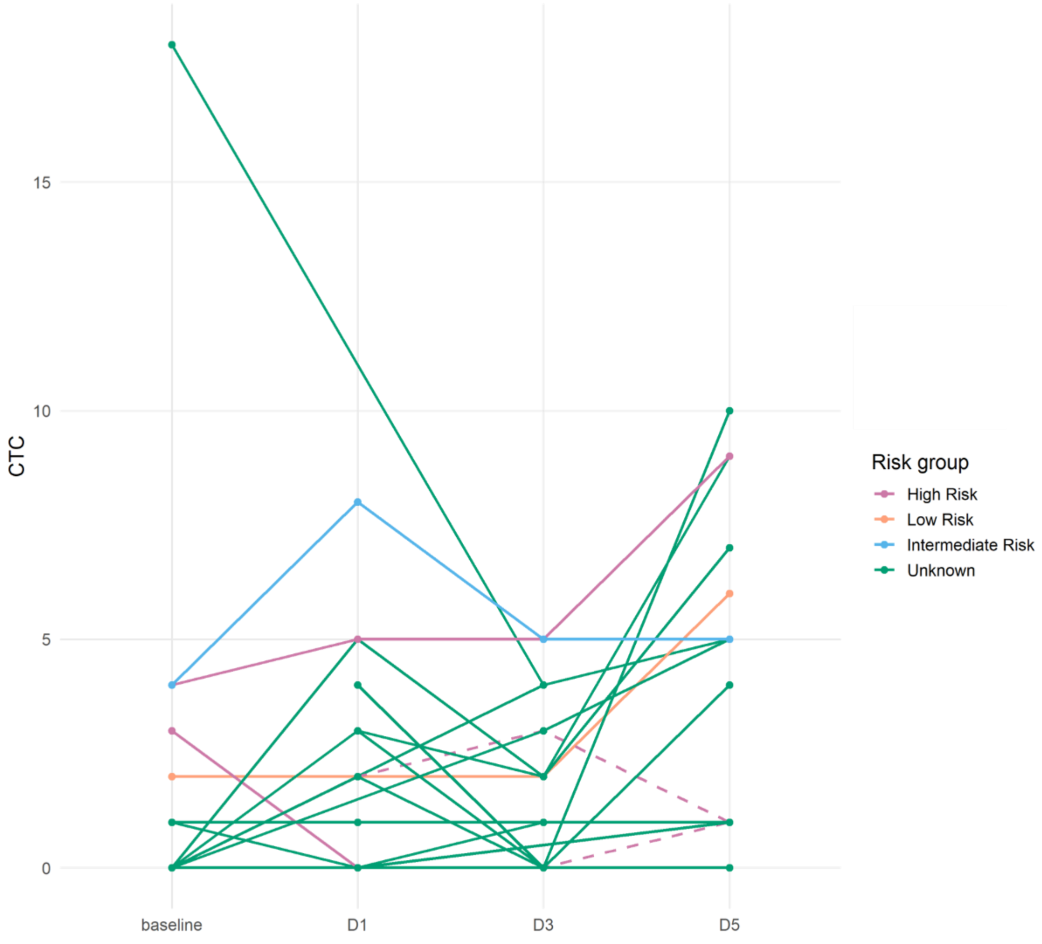

**References**


1. Amin, M.B.; Edge, S.B. *AJCC cancer staging manual*; springer: 2017.
2. Smit, K.N.; van Poppelen, N.M.; Vaarwater, J.; Verdijk, R.; van Marion, R.; Kalirai, H.; Coupland, S.E.; Thornton, S.; Farquhar, N.; Dubbink, H.J.; et al. Combined mutation and copy-number variation detection by targeted next-generation sequencing in uveal melanoma. *Mod Pathol* **2018**, *31*, 763-771, doi:10.1038/modpathol.2017.187.
